# Supplementary material for: Probiotics as Potential Tool to Mitigate Nucleotide Metabolism Alterations Induced by DiNP Dietary Exposure in Danio rerio
Source: Int J Mol Sci. 2024 Oct 17;25(20):11151. doi: 10.3390/ijms252011151 (PMC11508264; doi:10.3390/ijms252011151)
Supplement: Supplementary file 1 [file ijms-25-11151-s001.zip › ijms-3233014-supplementary.pdf]

SUPPLEMENTARY MATERIALS

Probiotics as Potential Tool to Mitigate Nucleotide Metabolism Alterations Induced by DiNP Dietary Exposure in *Danio rerio*

Christian Giommi [1,2], Francesca Maradonna [1,2], Claudia Ladisa [3], Hamid R. Habibi [3,†] and Oliana Carnevali [1,2,*,†]

[1] Department of Life and Environmental Sciences, Polytechnic University of Marche, 60131 Ancona, Italy; c.giommi@staff.univpm.it (C.G.); f.maradonna@staff.univpm.it (F.M.)

[2] INBB—Biostructures and Biosystems National Institute, 00136 Roma, Italy

[3] Department of Biological Sciences, University of Calgary, Calgary, AB T2N 1N4, Canada; cladisa@verschurencentre.ca (C.L.); habibi@ucalgary.ca (H.R.H.)

* Correspondence: o.carnevali@staff.univpm.it

† These authors contributed equally to this work.

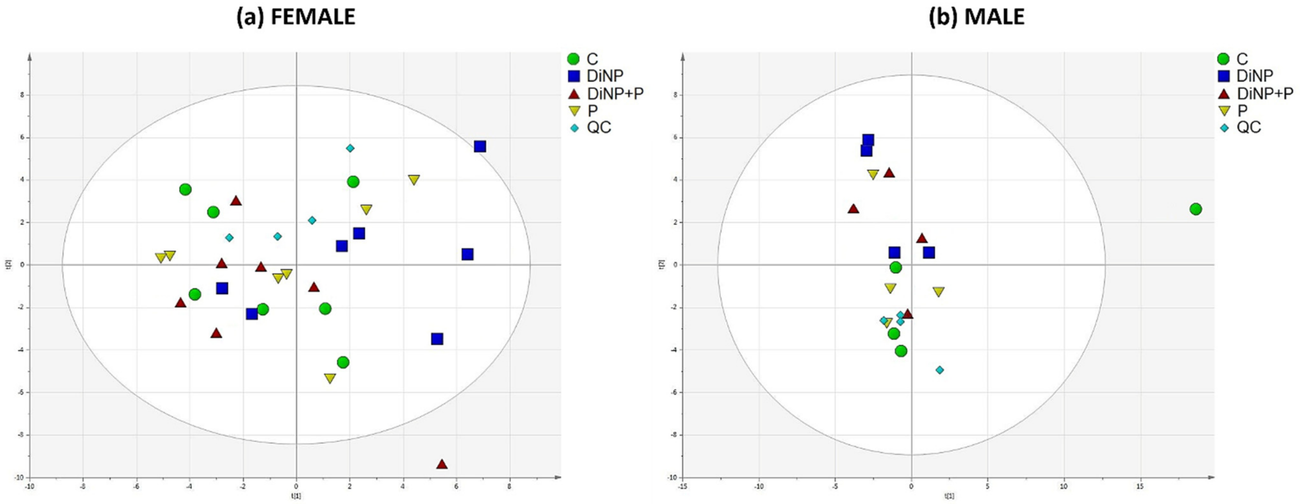

Supplementary Fig. 1 PCA score plot of all groups and QC (n = 4) in a) female (n = 7 per each group) and b) male (n = 4 per each group) livers, with C (green), DiNP (blue), DiNP+P (red) and P (yellow). Each symbol in the PCA represent a pool of at least 3 livers

(a) DiNP vs. C Female

Metabolites	VIP (25)
Citrulline	1.79
Guanosine monophosphate	1.71
Inosinic acid	1.68
Adenosine monophosphate	1.66
Pantothenic acid	1.65
Uridine 5'-monophosphate	1.63
D-Glucuronic acid	1.52
Fructose 6-phosphate	1.52
Azelaic acid	1.50
Uric acid	1.46
3-Hydroxybutyric acid	1.44
Cytidine monophosphate	1.43
Suberic acid	1.42
Hippuric acid	1.37
N-Acetylleucine	1.36
Itaconic acid	1.36
Guanosine	1.32
L-Cystathionine	1.29
Uridine	1.18
N-Acetyl-L-alanine	1.14
Adenine	1.13
D-Ribose	1.12
Inosine	1.09
Hypotaurine	1.08
L-Phenylalanine	1.03

(b) DiNP vs. C Male

Metabolites	VIP (30)
Pyridoxal	1.63
Uridine 5'-monophosphate	1.61
Cytidine monophosphate	1.48
Pyrrolidonecarboxylic acid	1.45
N-Acetylleucine	1.43
N-Acetyl-L-methionine	1.42
3-Hydroxybutyric acid	1.42
Adenine	1.42
Aminoadipic acid	1.36
Fumaric acid	1.35
L-Alanine	1.32
Succinic acid	1.30
Guanosine monophosphate	1.27
L-Glutamic acid	1.24
Acetoacetic acid	1.23
L-Malic acid	1.21
Hypoxanthine	1.21
Xanthine	1.20
L-Homoserine	1.19
Beta-Glycerophosphoric acid	1.18
L-Asparagine	1.18
Itaconic acid	1.17
Inosinic acid	1.15
Adenosine monophosphate	1.11
O-Phosphoethanolamine	1.09
Uracil	1.05
Alpha-D-Glucose	1.03
3-Hydroxymethylglutaric acid	1.03
Glutaric acid	1.01
L-Methionine	1.00

(c) DiNP+P vs. C Female

Metabolites	VIP (28)
Sucrose	2.19
L-Histidine	1.78
Adenosine monophosphate	1.77
3-Hydroxybutyric acid	1.73
Uric acid	1.65
Adenine	1.57
Uridine 5'-monophosphate	1.46
Glutathione	1.43
Guanosine monophosphate	1.40
Citrulline	1.38
Carnosine	1.37
L-Asparagine	1.35

(d) DiNP+P vs. C Male

Metabolites	VIP (27)
N-Acetyl-L-methionine	1.70
Uridine diphosphate-N-acetylglucosamine	1.69
Docosahexaenoic acid	1.63
Sucrose	1.54
N-Acetylleucine	1.51
L-Alanine	1.50
Pyridoxal	1.48
Adenine	1.41
Fumaric acid	1.37
Ornithine	1.31
L-Glutamic acid	1.23
Hypoxanthine	1.23

Hypotaurine	1.35
N-Acetyl-L-alanine	1.32
L-Glutamine	1.31
Adipic acid	1.30
L-Malic acid	1.29
Dihydroxyacetone phosphate	1.27
Cytidine monophosphate	1.25
Inosinic acid	1.24
Succinic acid	1.19
L-Glutamic acid	1.13
Alpha-D-Glucose	1.12
3-Hydroxymethylglutaric acid	1.08
Xanthine	1.03
Inosine	1.03
Fructose 6-phosphate	1.02
Allantoin	1.01

(e) P vs. C Female

**Metabolites**	**VIP (25)**
Hypotaurine	1.95
Fumaric acid	1.82
L-Histidine	1.80
Azelaic acid	1.72
Pantothenic acid	1.71
Suberic acid	1.67
Uric acid	1.55
Adenine	1.48
Xanthine	1.43
3-(2-Hydroxyphenyl)propanoic acid	1.42
Acetoacetic acid	1.41
Docosahexaenoic acid	1.39
L-Lysine	1.38
L-Aspartic acid	1.29
L-Tryptophan	1.27
L-Arginine	1.27
L-Phenylalanine	1.27
Ophthalmic acid	1.23
Uridine	1.22
Succinic acid	1.18
N-Acetyl-L-alanine	1.17
Adipic acid	1.16
D-Glucuronic acid	1.16
L-Homoserine	1.08
L-Asparagine	1.06

Ophthalmic acid	1.22
D-Ribose	1.21
Aminoadipic acid	1.20
Uracil	1.16
D-Ribose 5-phosphate	1.13
Pyrrolidonecarboxylic acid	1.12
L-Homoserine	1.08
L-Asparagine	1.07
Betaine	1.07
L-Cystathionine	1.05
Creatine	1.05
Allantoin	1.03
Beta-Glycerophosphoric acid	1.03
Uridine 5'-monophosphate	1.02
Acetoacetic acid	1.02

(f) P vs. C Male

**Metabolites**	**VIP (33)**
L-Homoserine	1.55
Docosahexaenoic acid	1.51
Fumaric acid	1.48
N-Acetyl-L-methionine	1.46
Hypoxanthine	1.41
N-Acetyl-L-alanine	1.33
Diaminopimelic acid	1.28
D-Glucuronic acid	1.26
3-(2-Hydroxyphenyl)propanoic acid	1.24
Pyrrolidonecarboxylic acid	1.24
Hippuric acid	1.23
Azelaic acid	1.20
L-Asparagine	1.17
Dihydroxyacetone phosphate	1.17
Uridine 5'-monophosphate	1.16
Cytidine monophosphate	1.16
Suberic acid	1.15
L-Phenylalanine	1.14
Pyridoxal	1.13
Ophthalmic acid	1.13
Acetoacetic acid	1.12
Beta-Glycerophosphoric acid	1.12
N-Acetylleucine	1.12
Guanosine	1.10
L-Lysine	1.08
L-Alanine	1.08
Uridine	1.08
L-Glutamic acid	1.06

		Aminoadipic acid	1.04
		D-Ribose	1.03
		Betaine	1.02
		O-Phosphoethanolamine	1.02
		3-Hydroxybutyric acid	1.02

Supplementary Table 1 VIPs > 1 found in PLS-DA-built models of DiNP vs. C, DiNP+P vs. C and P vs. C comparisons in a, c, e) female and b, d, f) male

(a) DiNP+P vs. DiNP Female	
**Metabolites**	**VIP (30)**
Citrulline	1.83
Fumaric acid	1.61
Adipic acid	1.61
Ophthalmic acid	1.59
L-Histidine	1.57
L-Aspartic acid	**1.54**
L-Tyrosine	1.52
Adenine	1.46
Acetoacetic acid	1.43
Hypotaurine	1.43
Alpha-D-Glucose	1.40
D-Ribose	1.38
L-Malic acid	1.34
L-Homoserine	1.33
3-Hydroxymethylglutaric acid	1.31
Inosine	1.31
Uridine	1.30
Inosinic acid	1.19
Fructose 6-phosphate	1.19
L-Lysine	1.18
Hippuric acid	1.16
L-Cystathionine	1.14
Guanosine monophosphate	1.13
Uracil	1.11
Itaconic acid	1.10
Xanthine	1.09
Pyrrolidonecarboxylic acid	1.05
Creatine	1.03
Adenosine monophosphate	1.01
L-Arginine	1.00

(b) DiNP+P vs. DiNP Male	
**Metabolites**	**VIP (28)**
L-Arginine	1.75
L-Methionine	1.71
L-Phenylalanine	1.69
L-Homoserine	1.65
Uridine diphosphate-N-acetylglucosamine	1.62
Betaine	1.61
L-Glutamine	1.60
Diaminopimelic acid	1.58
Docosahexaenoic acid	1.55
L-Tyrosine	1.54
L-Cystathionine	1.53
Xanthine	1.52
Pyridoxal	1.47
D-Ribose	1.47
3-Hydroxybutyric acid	1.46
L-Isoleucine	1.41
Hypotaurine	1.37
N-Acetyl-L-methionine	1.34
L-Malic acid	1.34
Ophthalmic acid	1.34
Dihydroxyacetone phosphate	1.33
L-Histidine	1.25
Itaconic acid	1.19
D-Glucuronic acid	1.19
Uric acid	1.14
Adenine	1.13
L-Serine	1.04
Guanosine	1.04

(c) DiNP+P vs. P Female	
**Metabolites**	**VIP (22)**
Sucrose	2.16
Azelaic acid	1.89
Suberic acid	1.89
Pantothenic acid	1.84
D-Glucuronic acid	1.67
Hypotaurine	1.61
L-Malic acid	1.58
Fumaric acid	1.55
Alpha-D-Glucose	1.51
L-Cystathionine	1.45
Carnosine	1.40
3-Hydroxymethylglutaric acid	1.40

(d) DiNP+P vs. P Male	
**Metabolites**	**VIP (22)**
Betaine	2.07
Ornithine	2.01
D-Ribose	1.92
Uridine diphosphate-N-acetylglucosamine	1.91
N-Acetyl-L-methionine	1.89
Creatine	1.82
Hypotaurine	1.76
Dihydroxyacetone phosphate	1.65
D-Glucuronic acid	1.57
Sucrose	1.52
Docosahexaenoic acid	1.41
Glutaric acid	1.37

L-Aspartic acid	1.31		Diaminopimelic acid	1.34
Acetoacetic acid	1.31		L-Alanine	1.33
L-Tryptophan	1.29		Azelaic acid	1.29
L-Glutamine	1.27		3-(2-Hydroxyphenyl)propanoic acid	1.25
L-Arginine	1.27		Fumaric acid	1.24
Citrulline	1.24		Adenine	1.20
Uracil	1.20		L-Homoserine	1.18
3-Hydroxybutyric acid	1.11		L-Phenylalanine	1.18
L-Tyrosine	1.08		N-Acetyl-L-alanine	1.13
Aminoadipic acid	1.02		Pyridoxal	1.13
			L-Glutamine	1.04

Supplementary Table 2 VIPs > 1 found in PLS-DA-built models of DiNP+P vs. DiNP and DiNP+P vs. P comparisons in a and c) female and b and d) male

(a)

DiNP vs. C	Fold Change	*p*-value
Guanosine monophosphate	0.382	0.016
Adenosine monophosphate	0.382	0.018
Inosinic acid	0.456	0.018
Hippuric acid	2.130	0.036

(b)

DiNP vs. C	Fold Change	*p*-value
Pyridoxal	0.325	0.003
N-Acetyl-L-methionine	3.499	0.011
Uridine 5'-monophosphate	0.161	0.016
L-Methionine	2.095	0.021
L-Alanine	4.873	0.033
Cytidine monophosphate	0.373	0.044
Xanthine	5.571	0.049
Glutaric acid	2.716	0.049

(c)

DiNP+P vs. C	Fold Change	*p*-value
Hypotaurine	4.528	0.003

(d)

DiNP+P vs. C	Fold Change	*p*-value
Docosahexaenoic acid	2.005	0.001
Hypoxanthine	4.277	0.043

(e)

P vs. C	Fold Change	*p*-value
Sucrose	2.649	0.0002
Uric acid	2.139	0.0007
3-Hydroxybutyric acid	2.226	0.013
Adenosine monophosphate	0.259	0.022

(f)

P vs. C	Fold Change	*p*-value
N-Acetyl-L-methionine	2.056	0.002
Docosahexaenoic acid	5.098	0.008
Uridine diphosphate-N-acetylglucosamine	2.505	0.013
Pyridoxal	0.398	0.017
L-Alanine	6.660	0.025
N-Acetylleucine	0.393	0.046

Supplementary Table 3. Volcano Plot analysis results showing metabolites changes in DiNP vs. C, DiNP+P vs. C and P vs. C comparisons in a, c, e) female and b, d, f) male. *p*-value and Fold Change were reported and metabolites with $p < 0.05$ were considered significant.

(a)

DiNP+P vs. DiNP	Fold Change	*p*-value
Hypotaurine	2.601	0.020
Ophthalmic acid	2.672	0.028
D-Ribose	0.454	0.046

(b)

DiNP+P vs. DiNP	Fold Change	*p*-value
L-Arginine	0.452	0.0003
L-Tyrosine	0.423	0.002
L-Phenylalanine	0.447	0.005
L-Methionine	0.473	0.008
L-Cystathionine	0.185	0.008
Diaminopimelic acid	0.358	0.014
L-Glutamine	0.259	0.015
Betaine	2.192	0.015
Dihydroxyacetone phosphate	0.286	0.022
Xanthine	0.132	0.024
L-Isoleucine	0.408	0.024
Pyridoxal	2.054	0.030

(c)

DiNP+P vs. P	Fold Change	*p*-value
Pantothenic acid	0.473	0.0005
Hypotaurine	2.323	0.021

(d)

DiNP+P vs. P	Fold Change	*p*-value
Betaine	2.732	0.0004
D-Ribose	3.298	0.004
Hypotaurine	3.408	0.014
Dihydroxyacetone phosphate	0.283	0.019

Supplementary Table 4 Volcano Plot analysis results showing metabolites changes in DiNP+P vs. DiNP and DiNP+P vs. P comparisons in a and c) female and b and d) male. *p*-value and Fold Change were reported and metabolites with $p < 0.05$ were considered significant

(a)

DiNP vs. C

**Pathway Name**	**Metabolites**	***p*-value**	**Impact**
Purine metabolism	AMP; IMP; GMP; Inosine; Urate; Guanosine; Adenine	0.016	0.259
Arginine biosynthesis	Citrulline	0.019	0.228
Pyrimidine metabolism	UMP; CMP; Uridine	0.023	0.109
Amino sugar and nucleotide sugar metabolism	D-Glucuronate; D-Fructose 6-phosphate	0.024	0.000
Ascorbate and aldarate metabolism	D-Glucuronate	0.028	0.625
Pentose and glucuronate interconversions	D-Glucuronate	0.028	0.182
Inositol phosphate metabolism	D-Glucuronate	0.028	0.000
Starch and sucrose metabolism	D-Fructose 6-phosphate	0.044	0.021

(b)

DiNP vs. C

**Pathway Name**	**Metabolites**	***p*-value**	**Impact**
Vitamin B6 metabolism	Pyridoxal	0.003	0.490
Pyrimidine metabolism	UMP; CMP; Uracil	0.020	0.185
Cysteine and methionine metabolism	Methionine	0.021	0.104
Aminoacyl-tRNA biosynthesis	Methionine; Asparagine; Alanine; Glutamate	0.021	0.000
Alanine, aspartate and glutamate metabolism	Asparagine; Alanine; Glutamate; Fumarate; Succinate	0.033	0.233
Selenocompound metabolism	Alanine	0.033	0.000

(c)

DiNP+P vs. C

**Pathway Name**	**Metabolites**	***p*-value**	**Impact**
Taurine and hypotaurine metabolism	Hypotaurine	0.003	0.400
Purine metabolism	Xanthine; Urate; Adenine	0.027	0.078
Aminoacyl-tRNA biosynthesis	Asparagine; Histidine; Phenylalanine; Arginine; Aspartate; Lysine; Tryptophan	0.047	0.000

(d)

DiNP+P vs. C

**Pathway Name**	**Metabolites**	***p*-value**	**Impact**
Arginine biosynthesis	Glutamate; Fumarate	3.51E-04	0.117
Citrate cycle (TCA cycle)	Fumarate	6.06E+00	0.030
Pyruvate metabolism	Fumarate	6.06E+00	0.000
Biosynthesis of unsaturated fatty acids	Docosahexaenoic acid	7.35E+00	0.000
D-Glutamine and D-glutamate metabolism	Gluatamate; 5-Oxo-D-proline	0.020	0.500
Vitamin B6 metabolism	Pyridoxal	0.041	0.490
Purine metabolism	Guanosine; Hypoxanthine	0.046	0.024

(e)

P vs. C

**Pathway Name**	**Metabolites**	***p*-value**	**Impact**
Galactose metabolism	Sucrose; Alpha-D-glucose	1.15E+00	0.068
Starch and sucrose metabolism	Sucrose; D-Fructose 6-phosphate	0.001	0.072
Fructose and mannose metabolism	Alpha-D-glucose, Glycerone phosphate	0.006	0.003
Glycolysis / Gluconeogenesis	Alpha-D-glucose, Glycerone phosphate	0.006	0.001

(f)

P vs. C

**Pathway Name**	**Metabolites**	***p*-value**	**Impact**
Histidine metabolism	Glutamate	7.37E+00	0.000
Glyoxylate and dicarboxylate metabolism	Glutamate	7.37E+00	0.000
Porphyrin and chlorophyll metabolism	Glutamate	7.37E+00	0.000
Nitrogen metabolism	Glutamate	7.37E+00	0.000

Synthesis and degradation of ketone bodies	3-Hydroxybutanoate	0.013	0.000
Butanoate metabolism	3-Hydroxybutanoate; Glutamate; Succinate	0.014	0.000
Glycerophospholipid metabolism	Glycerone phosphate	0.018	0.018
Glycerolipid metabolism	Glycerone phosphate	0.018	0.000
Inositol phosphate metabolism	Glycerone phosphate	0.018	0.000
Taurine and hypotaurine metabolism	Hypotaurine	0.026	0.400
Arginine biosynthesis	Glutamate; Citrulline; Glutamine	0.038	0.345

D-Glutamine and D-glutamate metabolism	Glutamate; 5-Oxo-D-proline	0.005	0.500
Arginine biosynthesis	Glutamate; Ornithine; Fumarate	0.007	0.178
Biosynthesis of unsaturated fatty acids	Docosaexaenoic acid	0.008	0.000
Starch and sucrose metabolism	Sucrose	0.009	0.052
Galactose metabolism	Sucrose	0.009	0.039
Amino sugar and nucleotide sugar metabolism	UDP-N-Acetyl-alpha-D-glucosamine	0.013	0.014
Vitamin B6 metabolism	Pyridoxal	0.017	0.490
Citrate cycle (TCA cycle)	Fumarate	0.022	0.030
Pyruvate metabolism	Fumarate	0.022	0.000
Alanine, aspartate and glutamate metabolism	Fumarate; Asparagine; Alanine; Glutamate	0.023	0.233
Aminoacyl-tRNA biosynthesis	Asparagine; Alanine; Glutamate	0.025	0.000
Selenocompound metabolism	Alanine	0.025	0.000
Glutathione metabolism	Glutamate; Ornithine	0.026	0.020
Arginine and proline metabolism	Glutamate; Ornithine; Creatine	0.031	0.224

Supplementary Table 5 MetPA tables showing main altered metabolic pathways found in DiNP vs. C, DiNP+P vs. C and P vs. C comparisons in a, c, e) female and b, d, f) male. Metabolites involved in the pathway, *p*-value and metabolites Impact inside the pathway were reported and pathways with $p < 0.05$ were considered significant

(a)

DiNP+P vs. DiNP

**Pathway Name**	**Metabolites**	***p*-value**	**Impact**
Taurine and hypotaurine metabolism	Hypotaurine	0.020	0.400
Alanine, aspartate and glutamate metabolism	Aspartate; Fumarate	0.030	0.219
Citrate cycle (TCA cycle)	Fumarate; Malate	0.034	0.074
Pyruvate metabolism	Fumarate; Malate	0.034	0.031
Nicotinate and nicotinamide metabolism	Aspartate	0.044	0.000
Tyrosine metabolism	Tyrosine; Fumarate; Acetoacetic acid	0.045	0.164
Pentose phosphate pathway	D-Ribose	0.046	0.000

(b)

DiNP+P vs. DiNP

**Pathway Name**	**Metabolites**	***p*-value**	**Impact**
Arginine and proline metabolism	Arginine	2.68E-01	0.055
Phenylalanine, tyrosine and tryptophan biosynthesis	Phenylalanine; Tyrosine	0.001	1.000
Phenylalanine metabolism	Phenylalanine; Tyrosine	0.001	0.357
Histidine metabolism	Histidine	0.001	0.224
beta-Alanine metabolism	Histidine	0.001	0.000
Tyrosine metabolism	Tyrosine	0.002	0.140
Ubiquinone and other terpenoid-quinone biosynthesis	Tyrosine	0.002	0.000
Aminoacyl-tRNA biosynthesis	Tyrosine; Histidine; Phenylalanine; Arginine; Glutamine	0.002	0.167
Arginine biosynthesis	Serine; Methionine; Isoleucine	0.004	0.076
Glyoxylate and dicarboxylate metabolism	Arginine; Glutamine	0.005	0.042
Glycine, serine and threonine metabolism	Glutamine; Malate; Serine	0.006	0.241
Cysteine and methionine metabolism	Serine; Betaine; Cystathionine	0.011	0.305
Alanine, aspartate and glutamate metabolism	Serine; Methionine; Cystathionine; Ophtalmic acid	0.015	0.133
Pyrimidine metabolism	Glutamine	0.015	0.000
D-Glutamine and D-glutamate metabolism	Glutamine	0.015	0.000
Nitrogen metabolism	Glutamine	0.015	0.000
Purine metabolism	Xanthine; Glutamine; Urate; Guanosine; Adenine	0.017	0.078
Glycerophospholipid metabolism	Glycerone phosphate	0.022	0.018
Fructose and mannose metabolism	Glycerone phosphate	0.022	0.003
Glycolysis / Gluconeogenesis	Glycerone phosphate	0.022	0.000
Glycerolipid metabolism	Glycerone phosphate	0.022	0.000
Inositol phosphate metabolism	Glycerone phosphate; Glucuronate	0.022	0.000
Valine, leucine and isoleucine degradation	Isoleucine	0.024	0.000

Valine, leucine and isoleucine biosynthesis	Isoleucine	0.024	0.000
Vitamin B6 metabolism	Pyridoxal	0.030	0.490

(c)

DiNP+P vs. P

**Pathway Name**	**Metabolites**	***p*-value**	**Impact**
Galactose metabolism	Sucrose; Alpha-D-glucose	0.001	0.068
Starch and sucrose metabolism	Sucrose	0.001	0.052
Amino sugar and nucleotide sugar metabolism	Glucuronate; Alpha-D-glucose	0.005	0.000
Citrate cycle (TCA cycle)	Malate; Fumarate	0.005	0.074
Pyruvate metabolism	Malate; Fumarate	0.005	0.031
Pantothenate and CoA biosynthesis	Pantothenate; Uracil	0.005	0.000
Glycolysis / Gluconeogenesis	Alpha-D-glucose	0.009	8.40E-04
Fructose and mannose metabolism	Alpha-D-glucose	0.009	0.000
Taurine and hypotaurine metabolism	Hypotaurine	0.021	0.400
Ascorbate and aldarate metabolism	Glucuronate	0.035	0.625
Pentose and glucuronate interconversions	Glucuronate	0.035	0.182
Inositol phosphate metabolism	Glucuronate	0.035	0.000
beta-Alanine metabolism	Aspartate; Carnosine; Uracil	0.044	0.062

(d)

DiNP+P vs. P

**Pathway Name**	**Metabolites**	***p*-value**	**Impact**
Glycine, serine and threonine metabolism	Betaine; Creatine	3.73E+00	0.048
Arginine and proline metabolism	Creatine; Ornithine	6.08E+00	0.139
Glutathione metabolism	Ornithine	0.002	0.000
Pentose phosphate pathway	D-Ribose	0.004	0.000
Inositol phosphate metabolism	Glycerone phosphate, Glucuronate	0.011	0.000
Taurine and hypotaurine metabolism	Hypotaurine	0.014	0.400
Starch and sucrose metabolism	Sucrose	0.017	0.052
Galactose metabolism	Sucrose	0.017	0.039
Glycerophospholipid metabolism	Glycerone phosphate	0.019	0.018
Fructose and mannose metabolism	Glycerone phosphate	0.019	0.003
Glycolysis / Gluconeogenesis	Glycerone phosphate	0.019	0.000
Glycerolipid metabolism	Glycerone phosphate	0.019	0.000
Amino sugar and nucleotide sugar metabolism	Glucuronate; UDP-N-Acetyl-alpha-D-glucosamine	0.024	0.014
Ascorbate and aldarate metabolism	Glucuronate	0.033	0.625
Pentose and glucuronate interconversions	Glucuronate	0.033	0.182
Arginine biosynthesis	Ornithine; Glutamate; Fumarate	0.037	0.061

Supplementary Table 6 MetPA tables showing main altered metabolic pathways found in DiNP+P vs. DiNP and DiNP+P vs. P comparisons in a and c) female and b and d) male. Metabolites involved in the pathway, *p*-value and metabolites Impact inside the pathway were reported and pathways with $p < 0.05$ were considered significant
